# Deep learning image enhancement for confident diagnosis of TMJ osteoarthritis in zero-TE MR imaging

**DOI:** 10.1093/dmfr/twae063

**Published:** 2025-02-24

**Authors:** Chena Lee, Joonsung Lee, Sagar Mandava, Maggie Fung, Yoon Joo Choi, Kug Jin Jeon, Sang-Sun Han

**Affiliations:** Department of Oral and Maxillofacial Radiology, Yonsei University College of Dentistry, Seoul, 03722, Republic of Korea; Institute for Innovative in Digital Healthcare, Seoul, 03722, Republic of Korea; GE HealthCare, Seoul, 04637, Republic of Korea; GE HealthCare, Atlanta, GA, 30188, United States; GE HealthCare, New York, NY, 10032, United States; Department of Oral and Maxillofacial Radiology, Yonsei University College of Dentistry, Seoul, 03722, Republic of Korea; Department of Oral and Maxillofacial Radiology, Yonsei University College of Dentistry, Seoul, 03722, Republic of Korea; Department of Oral and Maxillofacial Radiology, Yonsei University College of Dentistry, Seoul, 03722, Republic of Korea; Institute for Innovative in Digital Healthcare, Seoul, 03722, Republic of Korea; Oral Science Research Center, Yonsei University College of Dentistry, Seoul, 03722, Republic of Korea

**Keywords:** convolutional neural network, digital radiography, MRI, temporomandibular joint disorders

## Abstract

**Objectives:**

This study aimed to evaluate the effectiveness of deep learning method for denoising and artefact reduction (AR) in zero echo time MRI (ZTE-MRI). Also, clinical applicability was evaluated by comparing image diagnosis to the temporomandibular joint (TMJ) cone-beam CT (CBCT).

**Methods:**

CBCT and routine ZTE-MRI data were collected for 30 patients, along with an additional ZTE-MRI obtained with reduced scan time. Scan time-reduced image sets were processed into denoised and AR images based on a deep learning technique. The image quality of the routine sequence, denoised, and AR image sets was compared quantitatively using the signal-to-noise ratio (SNR) and qualitatively using a 3-point grading system (0: poor, 1: good, 2: excellent). The presence of osteoarthritis was assessed in each imaging protocol. Diagnostic accuracy of each protocol was compared against the CBCT results, which served as the reference standard. The SNR and the qualitative scores were compared using analysis of variance test and Kruskal-Wallis test, respectively. The diagnostic accuracy was assessed using Cohen’s κ (<0.5 = poor; 0.5 to <0.75 = moderate; 0.75 to <0.9 = good; ≥0.9 = excellent).

**Results:**

Both the denoised and AR protocols resulted in significantly enhanced SNR compared to the routine protocol, with the AR protocol showing a higher SNR than the denoised one. The qualitative assessment also showed highest grade in AR protocol with statistical significance. The osteoarthritis diagnosis showed enhanced agreement with CBCT in denoised (κ = 0.928) and AR images (κ = 0.929) than routine images (κ = 0.707).

**Conclusions:**

A newly developed deep learning technique for both denoising and artefact reduction in ZTE-MRI presented clinical usefulness. Specifically, AR protocol showed significantly improved image quality and comparable diagnostic accuracy comparable to CBCT. It can be expected that this novel technique would help overcome the current limitation of ZTE-MRI for replacing CBCT in bone imaging of TMJ.

## Introduction

Deep learning (DL) technology is widely used in various medical imaging fields, driven by its rapid development. Among these, the technology being applied most rapidly in the medical imaging field, with image quality enhancement benefiting the most from it.^1–5^ In the case of MRI, tedious scan times have always been a limitation, and image improvement using DL is gradually being introduced as a solution.^4^^,^^5^ DL was initially introduced in slice-by-slice imaging, known as 2D sequences. DL-based reconstruction using undersampled MRI offers a new approach to reducing examination times while maintaining image quality.^6^^,^^7^

Bone MRI is a sequence that is expected to be very useful in the medical and dental field, which focuses on hard tissues.^8–10^ However, limitations include that the imaging time is considerably longer than that of CT. Also, the noise level and artefacts on tissue interface are abundant compared to CT images, so the clinician may be confused and unfamiliar with the image contrast and pattern during the interpretation.^8^^,^^9^ Previous studies have also reported that the accuracy of bone description in the image is comparable to that of CT, but interpretation can be challenging due to unfamiliar image quality.^8^^,^^9^

To solve this problem, DL research on MRI transformation using CT and CBCT (cone-beam CT) images has been conducted.^9^ In this study, image-to-image transformation was attempted using a DL model. According to the research, bone MRI could be effectively synthesized to show a similar style to CT. However, since clinical MR image-CT image pairs were used as training data, incomplete registration between the 2 images acted as a limitation. Thus, the resultant image was blurry, which served as a limitation of the study. Accordingly, it is believed that image improvement through the application of a DL model at the k-space level is essential.^9^^,^^11^

DL image quality improvement based on k-space has recently been attempted for several sequences. Verification was conducted through clinical image quality evaluation for various body parts in 3-dimensional T1 and T2 sequences.^4–6^ However, to the authors’ knowledge, no study has used bone MRI with a ZTE sequence for temporomandibular joint (TMJ) diagnosis. Therefore, this study aims to evaluate the effectiveness of image enhancement through a DL process of denoised and artefact-reduced protocols on zero echo time MRI (ZTE-MRI). Also, clinical accuracy was evaluated by comparing image interpretation results to the TMJ CBCT image, which is the current standard diagnostic imaging modality for TMJ osteoarthritis detection.

## Methods

**Figure 1.**
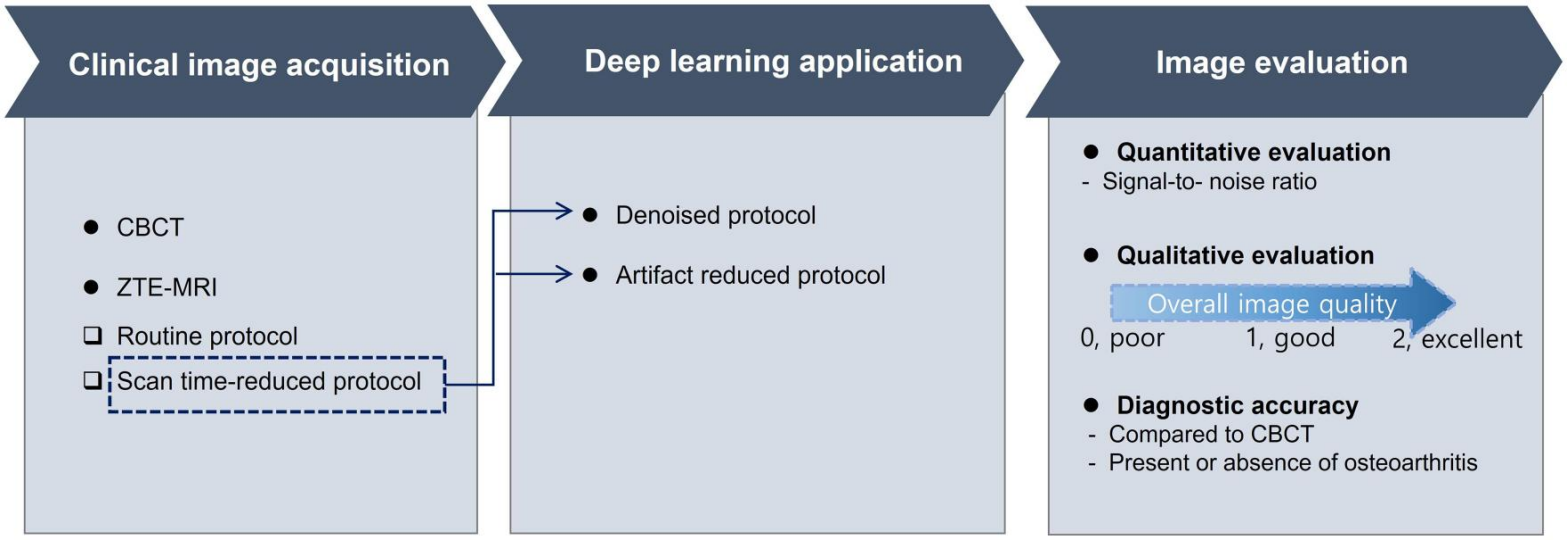
Schematic workflow of the overall study design.

The schematic study design is described in Figure 1.

### Clinical image acquisition

Thirty patients who underwent both CBCT and MRI examinations between January 1, 2023 and August 31, 2023, for TMJ evaluation were selected. For ZTE sequence in MRI, in addition to routine protocol, scan time-reduced sequence was additionally obtained. The study was conducted under the approval of the institutional review board of Yonsei University Dental Hospital (no. 2-2021-0027).

The imaging condition of CBCT was as follows: Alphard 3030 device (Asahi Roentgen Ind., Co. Ltd, Kyoto, Japan); tube voltage, 90 kVp; tube current, 8 mA; exposure time, 17 s; FOV (Field-of-view), 15 × 15 cm. The ZTE-MRI data were obtained using the following parameters: SIGNA™ Pioneer (GE HealthCare, Waukesha, WI, USA) with a 21-channel head coil; Isotropic 3-dimensional proton-density weighted ZTE scans, 31.25 kHz; NEX, 1 and 2; FOV, 180 × 180 mm; acquisition matrix, 260 × 260, slice thickness, 1.0 mm. For routine and scan time-reduced protocol, scan time was ∼5 min and ∼2 min, respectively, by adjusting NEX.

### Deep learning application

For the image sets obtained with scan time-reduction, an industry-developed DL algorithm, the prototype version of AIR™ Recon DL for ZTE, was applied. Using the DL Recon, scan time-reduced image set was processed into (a) denoised image and (b) artefact reduced (AR) and denoised image. Conventional ZTE-MRI presents white and dark banding artefact, especially on the border of tissue interface. In the present study, DL protocol with denoised image was defined as the denoised protocol and that with AR and denoised image was defined as the AR protocol.

The DL algorithm used in this study is based on reconstruction that takes raw k-space data as input and generates high-fidelity images as output.^12–15^ The AIR™ Recon DL pipeline is a deep convolutional network (CNN) system designed to process raw, complex-valued imaging data. It consists of approximately 4.4 million trainable parameters across around 10 000 kernels, making it capable of handling all relevant MRI image sizes. The CNN was trained with the pairs of near-perfect and conventional MR images. The near-perfect training data, consisting of the original high-resolution images, had minimal ringing and very low levels of noise. To create the conventional training data, these high-quality images were processed using established methods, resulting in lower-resolution versions that included more truncation artefacts and higher noise levels. Training was performed in a single epoch of the training database with loss function of the ADAM optimizer.

### Image evaluation

#### Quantitative evaluation

Thirty image sets of 3 different protocols—routine, denoised, and AR—were compared in terms of signal-to-noise ratio (SNR). For SNR analysis, the coronal image was selected in the mid-plane, where it best showed both mandibular condyles based on the agreement of 2 radiologists. The SNR was calculated by dividing the mean signal by the standard deviation of noise. The noise levels were obtained using a combination of discrete wavelet transform methods, and the edges were removed by the algorithm described in previous studies.^16^^,^^17^

#### Qualitative evaluation

The image sets from respective protocols were evaluated using a 3-scale grading system. The grades were 0, poor quality that cannot be interpreted; 1, good quality of readable image; 2, excellent quality that helps confident interpretation. All axial sectional views were thoroughly evaluated by 2 evaluators respectively. The imaging protocol information was blinded and evaluators were calibrated for scoring before the evaluation was conducted.

#### Diagnostic accuracy

For a total of 60 TMJs, 3 oral and maxillofacial radiologists reached a consensus on the presence or absence of osteoarthritis in the TMJ based on coronal and sagittal views of CBCT. This was considered as the reference standard. Then, 2 evaluators independently assessed the presence or absence of osteoarthritis based on the ZTE-MRI data of the same joint. Three different ZTE-MRI protocols were evaluated in a random sequential manner. Before the evaluators conducted the test, the diagnosis criteria were calibrated based on the previous studies.^18^

#### Statistical test

Statistical test was performed using GraphPad Prism version 10.0.0 for Windows, GraphPad Software, Boston, Massachusetts USA, www.graphpad.com. SNR among each protocol was compared using the analysis of variance (ANOVA) test with 95% confidential interval. The scores of qualitative image grading system were compared using Kruskal-Wallis test with 95% confidential interval. The degree of agreement of the ZTE-MRI result with that of CBCT was analysed through the Cohen κ (<0.5 = poor; 0.5 to <0.75 = moderate; 0.75 to <0.9 = good; ≥0.9 = excellent).

## Results

**Figure 2.**
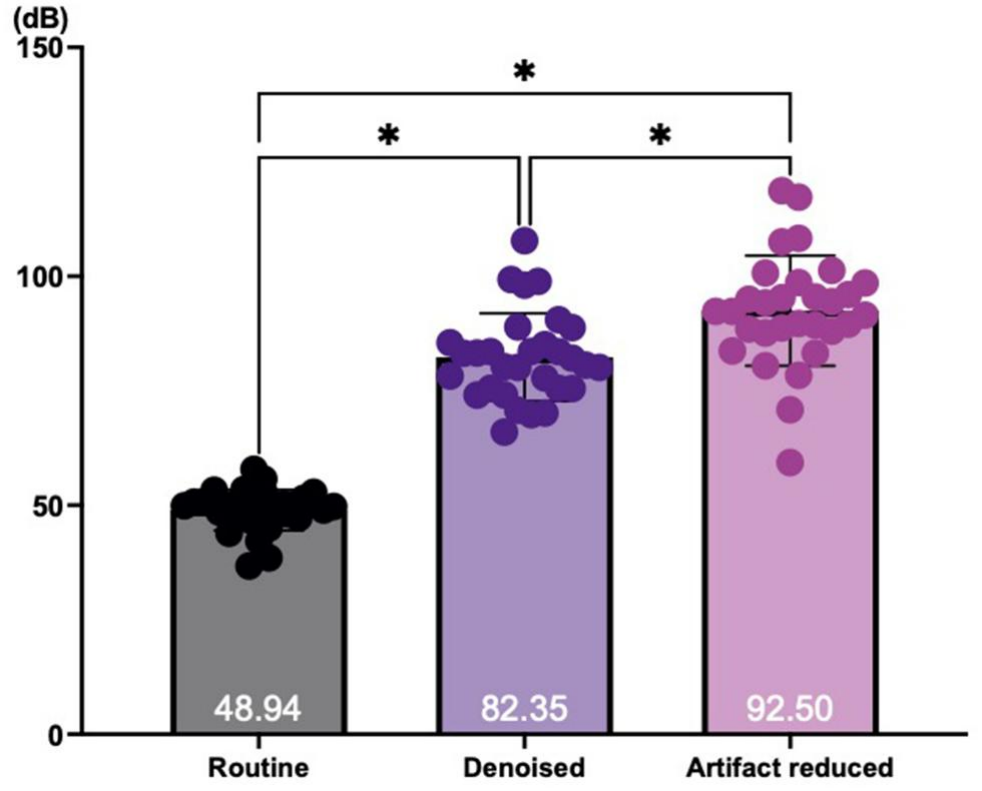
Signal-to-noise ratio comparisons (ANOVA, *P < 0.05). Abbreviation: ANOVA = analysis of variance.

**Figure 3.**
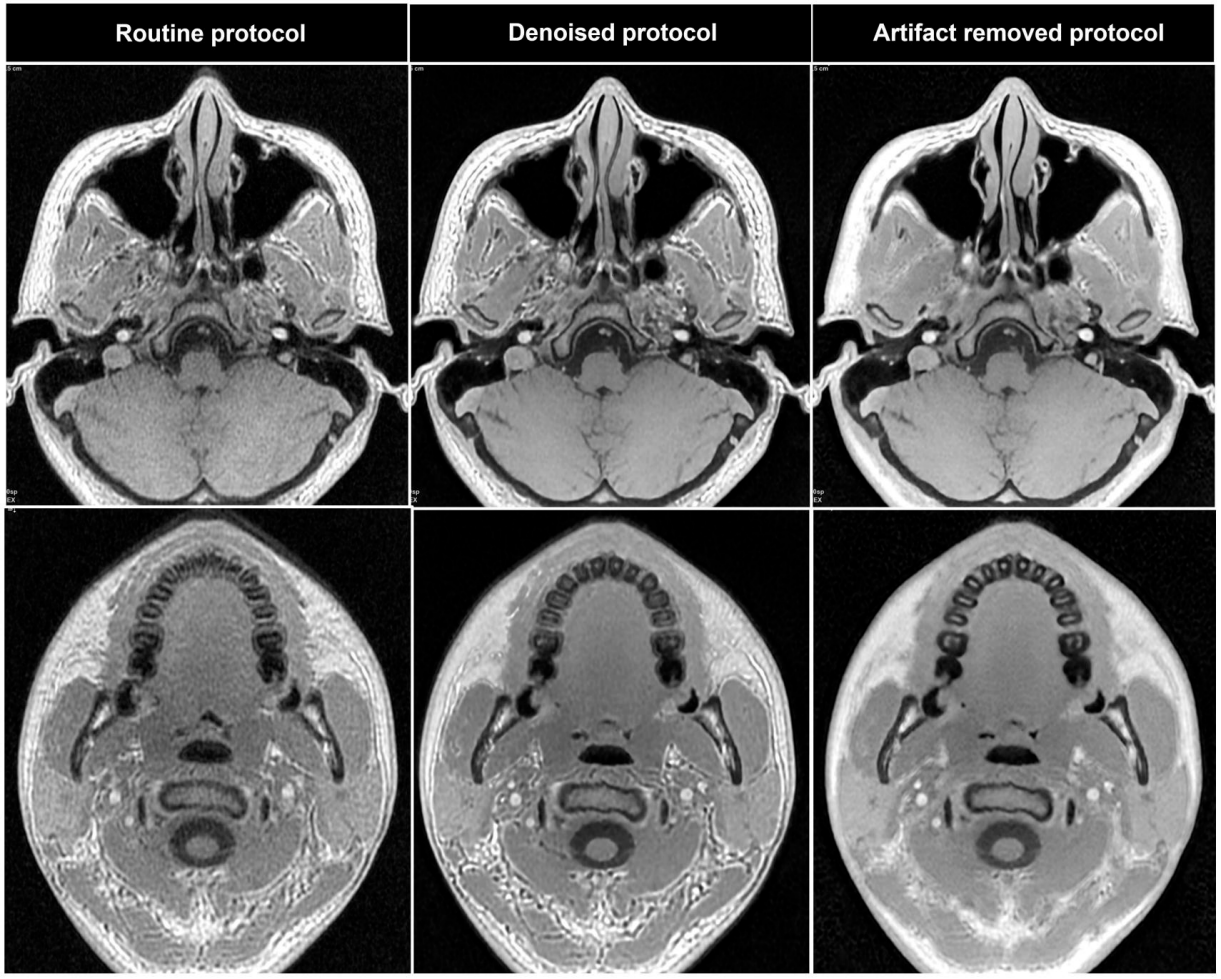
Overall image quality of routine, denoised, and artefact-reduced protocol.

**Figure 4.**
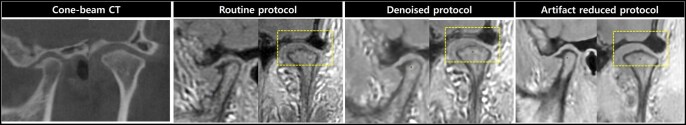
Representative joint image with presence of osteoarthritis in CBCT, ZTE-MRI of routine, denoised, and AR image. Note that routine and denoised protocol have artefacts within joint space which may cause mis-diagnosis while the osseous part in AR protocol is presented clearly in AR protocol comparable to CBCT (dotted box). Abbreviations: CBCT = cone-beam CT; ZTE-MRI = zero echo time MRI; AR = artefact reduced.

Both denoised and AR protocols generated images with higher SNR with significance compared to routine protocol (Figure 2). Additionally, between the DL protocols, AR protocol showed higher SNR with significance. The qualitative assessment also showed that the highest mean score in the grading system was for AR images, followed by denoised and routine images, with no statistical significance between them (Table 1, Figure 3). For diagnostic performance, denoised and AR protocol revealed “excellent agreement (κ = 0.928 and 0.929)” with CBCT. The routine protocol showed moderate agreement (κ = 0.707) with CBCT (Table 2, Figure 4).

**Table 1.** Mean and standard deviation of the overall image grade.

Routine	Denoised	Artefact reduced	*P*-value^a^
1.103 ± 0.409^b^	1.586 ± 0.501^b^	1.828 ± 0.384^c^	<0.05

a Kruskal-Wallis test, confidential interval of 95%.

b is significantly different from ^c^

**Table 2.** Agreement of each ZTE protocol with CBCT in Cohen’s kappa (κ) coefficient.^a^

Routine protocol	Deep learning protocol	
	Denoised protocol	Artefact reduced
0.707	0.928	0.929

Abbreviations: ZTE-MRI = zero echo time MRI; CBCT = cone-beam CT.

a <0.5 = poor; 0.5 to <0.75 = moderate; 0.75 to <0.9 = good; ≥0.9 = excellent.

## Discussion

ZTE-MRI introduced bone image with clinical feasibility; however, its characteristic feature of high noise and the artefact formed at the interface of the tissue was a major issue that required resolution.^8–10^ Due to the recent advancement of DL image enhancement, it was possible to reduce noise and eliminate artefacts in MRI images, and this study was conducted to ensure that the DL-processed image might actually help radiologists to diagnose with more confidence.^6^ The DL-based denoised and AR protocol presented better image quality compared to the routine protocol, though DL protocols were based on the image set of reduced scan time. This result was confirmed in both quantitative and qualitative methods. A diagnostic support of DL was also significant in that it showed closer agreement with the reference. In other words, scan time can be reduced without sacrificing either image quality or diagnostic accuracy.

The interesting point of the current study was that the DL-based denoising alone brought the diagnostic accuracy closer to the reference, but there was no statistically significant improvement in the overall image quality score when radiologists graded the entire sequence. However, in AR images, both diagnostic accuracy and image quality showed a statistically significant improvement. It was obvious that the white and black lines forming at tissue interfaces were reduced in AR images. It was thought that this significantly contributed to the radiologists recognizing the ZTE-MRI as more similar to CBCT and becoming familiar with the image itself. In addition, reduction of artefacts actually helped readers to determine osteoarthritis with more ease since with the presence of artefacts in a small complex region, specifically joint space, radiologists need more concentration to distinguish between real bone particles and the artefacts. The difficulty in making diagnostic decision based on ZTE-MRI has been mentioned in previous studies. ZTE images were unfamiliar to readers, leading to high levels of evaluation fatigue.^8–10^ These studies concluded that the image characteristics of ZTE-MRI did not affect the diagnostic accuracy, but were difficult to interpret. In addition, it can be thought that it may affect the diagnosis for non-imaging experts. Therefore, the introduction of ZTE-MRI with AR protocol showing closer texture to the CT image is a key point that should be noted in this study.

There were many previous studies conducted for image quality enhancement in conventional sequence of T1- or T2-weighted images through DL techniques.^1^^,^^5^^,^^6^ For now, commercialized products of DL image enhancement have been widely used for conventional sequences. Most of the studies focused on enhancement in quality of image which was based on undersampling with low SNR.^5^^,^^6^ The current study also concentrated on the same concept that undersampled data with shortened acquisition time could be reconstructed into better image quality. However, this study was advanced to the next step of removing the white and dark line artefacts, which have been accepted as specific but annoying image features of ZTE-MRI. These types of artefacts caused radiologists to be distracted by soft tissues rather than focusing on hard tissues, even though the purpose of ZTE images is bony evaluation. Moreover, to the authors’ knowledge, this is the first study to apply DL processing for artefact elimination in ZTE-MRI.

In particular, the current study is significant as the scan time was reduced to half that of the routine sequence. The relatively long scan time has been a limitation preventing ZTE-MRI from replacing CT or CBCT. In the current study, the scan time was reduced by adjusting the number of excitations (NEX), a commonly used strategy for shortening scan times. However, this method is also commonly used to increase the SNR of the image.^6^ Thus, decreasing NEX inevitably led to lower image quality with higher noise. The denoising method used in this study greatly enhanced the SNR of the ZTE-MRI. Also, this affected the diagnostic accuracy in a positive way. Since the diagnosis of TMJ arthritis relies on minute changes in the small osseous structures of the joint, denoising was thought to be very helpful in enabling radiologists to make confident judgments.

Although radiation exposure due to CT or CBCT cannot be disregarded, bone imaging is essential for TMJ pathology diagnosis. Specifically, CBCT has been a useful modality for identifying fine pathologic changes in the osseous structures of the joints. And, multiple CBCT examination for interval check-up is commonly conducted in clinical condition due to joint morphological changes that may also cause other orofacial symptoms such as changes in occlusion.^19^ ZTE-MRI was depicted as good replacement for hard tissue pathology diagnosis instead of CT or CBCT.^8–10^^,^^20^ However, for clinical application, there have been several challenges to overcome. The major issues were long scan time and imaging traits of high noise. Thanks to recent advancements in deep learning techniques, these challenges are nearly being overcome, and it is expected that ZTE-MRI will gradually be used as a protocol that can replace CT.

This study has several limitations, including the lack of more detailed joint evaluation due to the limited sample size. In this study, joint pathology was simply divided into 2 groups: presence or absence of osteoarthritis. However, the status of arthritis can be described in more detailed stages, even with minute differences in the trabecular bone pattern.^18^^,^^21^ In future studies, larger sample sizes representing various stages of osteoarthritis could be included to enhance the clinical utilization of the DL protocol. The ultimate goal of this study is to promote the use of ZTE-MRI as an alternative to CT or CBCT, particularly for patients currently undergoing both CBCT/CT and MRI examinations.

In conclusion, a newly developed DL technique for denoising and artefact reduction in ZTE-MRI has been evaluated for its clinical usefulness. Due to the improved image quality, this method may help radiologists diagnose TMJ arthritis with accuracy comparable to that achieved with CBCT, the current reference imaging modality for TMJ bone imaging.
